# The Response to Enthusiasm

**Published:** 1920-02-21

**Authors:** 


### The Response to Enthusiasm.

The Leicester Royal Infirmary benefits annually from a matinée at the Palace Theatre. The cause is good, but without the enthusiasm of a hard-working Matinée Committee support would be lukewarm. Sir Oswald Stoll gives the free use of the theatre, the staff, and orchestra, and the players give their services, and the Matinée Committee arranges the programme and disposes of the tickets, etc. Net receipts from donations, sale of tickets, programmes, fruit and flowers, and proceeds of advertisements amount this year to £564. Interest allowed by bank, an item denoting a sympathetic banker, adds £1 8s., making the total receipts £565 odd. On the other side of the account the expenditure is "nil."

At a recent meeting of the Matinée Committee, Mr. Councillor Baum presiding, Mr. D. J. Williams (Hon. Treasurer) had the pleasure of handing to Mr. T. Fielding Johnson, for the chairman of the infirmary, a cheque for this amount. Opportunity was taken by the infirmary chairman to acknowledge the public-spirited work of the Matinée Committee and the great help which had been given to the infirmary for several years. The present was the ninth matinée which had been organised, and the Matinée Committee's generous work has benefited the infirmary by £2,300.

